# The Cadenza lyric intelligibility prediction (CLIP) dataset

**DOI:** 10.1016/j.dib.2026.112466

**Published:** 2026-01-14

**Authors:** Gerardo Roa-Dabike, Trevor J. Cox, Jon P. Barker, Bruno M. Fazenda, Simone Graetzer, Rebecca R. Vos, Michael A. Akeroyd, Jennifer Firth, William M. Whitmer, Scott Bannister, Alinka Greasley

**Affiliations:** a School of Computer Science, University of Sheffield, UK; b Acoustics Research Centre, University of Salford, UK; c Hearing Sciences, Mental Health and Clinical Neurosciences, School of Medicine, University of Nottingham, UK; d School of Music, University of Leeds, UK

**Keywords:** Music, Singing, English, MIR, Deep learning, Machine learning, Hearing, Hearing loss

## Abstract

This paper presents CLIP, a dataset of 11,072 popular western music signals sourced from independent artists, accompanied by ground truth lyrics, and lyric intelligibility scores from listening tests. The dataset is designed to facilitate music information retrieval (MIR) research using machine learning. It was created to allow the development of algorithms to predict lyric intelligibility for the Cadenza ICASSP 2026 Signal Processing Grand Challenge. Currently, it is the only publicly available large-scale dataset for such a task. The music was sourced from the Free Music Archive (FMA) dataset and is unlikely to be familiar to listeners. We excluded tracks whose license did not allow derivative works and those that did not have English singing. Ground truth transcriptions were generated by seven native English speakers, resulting in 3700 excerpts of 5 to 10 words each from 1452 different songs. A hearing loss simulation was also applied to the stereo audio. This resulted in 11,100 music signals with no, mild or moderate hearing loss. This was done so more diverse hearing is represented in the dataset. Human transcriptions were then collected via an online listening experiment. Participants self-reported as having normal-hearing and being native English speakers. They listened to each music signal twice before transcribing each line. Final intelligibility scores were the ratio of matching words between the listening test responses and the ground truth transcriptions. The final dataset consists of audio, ground truth lyrics, intelligibility scores and associated metadata.

Specifications Table

**Table:** 

Subject	Data Science: Applied Machine Learning
Specific subject area	Developing Music Information Retrieval (MIR) algorithms for predicting lyric intelligibility where some music has been processed by a hearing loss simulation.
Type of data	Digital audio files in FLAC format Metadata in JSON format
Data collection	The dataset provides music excerpts paired with ground truth transcriptions; intelligibility scores obtained through listening tests, and metadata including the simulated hearing loss severity. The music excerpts were derived from the Free Music Archive (FMA) dataset [1], which contains >106,000 songs but without ground truth lyrics. A pool of over 17,000 popular Western music tracks was extracted from the FMA dataset by choosing those with English vocals and licenses that allow derivative works. Ground truth transcriptions were generated by native English speakers using Label Studio [2], resulting in 3700 excerpts from 1452 songs. Each excerpt was then processed using a hearing loss simulator applying different audiograms. This produced a total of 11,100 music signals with no, mild or moderate hearing loss. Using Prolific (www.prolific.com), we recruited 111 native English-speaking participants with self-reported normal hearing. They transcribed each music signal after hearing it twice. The dataset is split into training, validation, and evaluation sets, disjoint by artist and audiogram.
Data source location	Institution: University of Salford City/Town/Region: Salford Country: UK*.*
Data accessibility	Repository name: Zenodo Data identification number: 10.5281/zenodo.17950664 Direct URL to data: https://zenodo.org/records/17950664 Dataset freely available and has a Creative Commons Attribution-Share Alike 4.0 International License.
Related research article	N/A*.*

## 1 Value of the Data

• Music Information Retrieval (MIR) develops and applies computer algorithms to analyze and understand music. The CLIP dataset was created to allow the development of MIR algorithms that predict lyric intelligibility for popular Western music sung in English. It might also be applied to other MIR tasks such as Automatic Lyric Transcription (ALT).
• CLIP was developed because there were no datasets large enough to allow deep learning algorithms to be developed for Lyric Intelligibility Prediction. This is the first large dataset with song excerpts, ground truth lyrics and intelligibility scores from listening test.
• Most datasets used to develop machine listening algorithms do not consider hearing diversity. Yet non-normal hearing is very common, for example, one in three adults in the UK is deaf, has hearing loss or tinnitus has hearing loss or tinnitus. To address this, two-thirds of the audio in the CLIP dataset were degraded using a hearing loss simulator, so intelligibility scores considered a wider population.

## 2 Background

The Cadenza project aims to improve music accessibility for people with hearing loss through a series of machine learning challenges. According to the World Health Organization, >1.5 billion people worldwide currently experience hearing loss, a number projected to rise to 2.5 billion by 2050 [3].

Understanding lyrics is an important part of enjoying music [4]. However, people with hearing loss can struggle to perceive lyrics clearly and effortlessly [5]. Fine and Ginsborg [4] define intelligibility as “the extent to which the speaker’s or singer’s message can be understood by the listener”, whereas the Cadenza’s sensory panel of hearing aid users described it as “how clearly and effortlessly the words in the music can be heard.” Although different, both definitions focus on the clarity of the message conveyed in music.

This motivated the Cadenza project team to run a machine learning challenge focused on predicting lyric intelligibility. In spoken language technologies, the development of automatic intelligibility metrics has driven advances in tasks such as speech enhancement. Similarly, reliable metrics for lyric intelligibility could underpin systems that improve music. The intelligibility could be monitored during recording, production and reproduction and adjustments made to the music using signal processing or machine learning. Metrics could also provide insights into how lyrics are perceived by people with hearing loss.

## 3 Data Description

The dataset is a combination of audio signals and metadata including the associated ground truth lyrics and intelligibility scores. It is provided with predefined splits for training, validation, and evaluation for machine learning. Audio signals and metadata are packaged in tar.gz files, with the version number indicated in the filename. Each package is intended to be unpacked into the same directory.

For example, in Version 1 of the dataset, the packages are named as follows:

Training: cadenza_clip1_data.train.v1.0.tar.gz
Validation: cadenza_clip1_data.valid.v1.0.tar.gz
Evaluation: cadenza_clip1_data.eval.v1.0.tar.gz

**Fig. 1:**
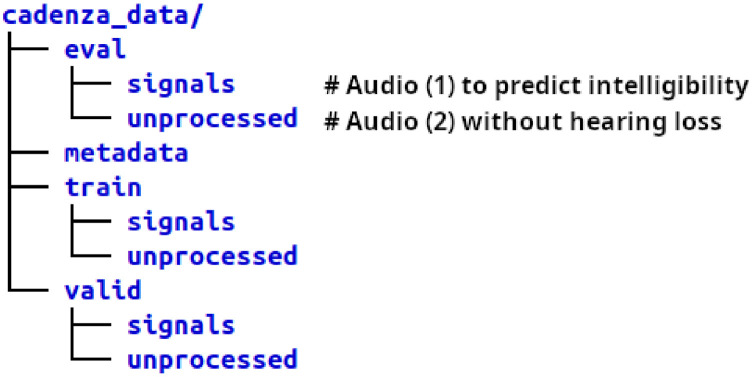
Files structure after unpacking the tar.gz files.

After extracting all packages, the file structure is as shown in Fig. 1. The train, validation, and evaluation directories contain both processed and unprocessed audio signals, while the metadata directory contains a separate JSON file for each split. A manifest file per split is also provided with the checksum for each audio file. All audio files are stored in 16-bit FLAC format with a sampling frequency of 44,100 Hz.

The metadata directory contains a JSON file per split with the details for each excerpt (see Table 1)

**Table 1:** Description of JSON metadata.

Field name	Description	Example
signal	Unique identifier for the audio file.	“785b38c0c7c95596841b774a”
fma	Track ID from the Free Music Archive (FMA) dataset.	“126,666″
original_prompt	Ground-truth sentence as transcribed by annotators (before text normalization)	“sorry I let you down if I let you down”
prompt	Normalized version of original_prompt (used as ground truth for scoring)	"sorry i let you down if i let you down"
prompt_pronunciation	Phonemic transcription of the prompt using the BEEP pronunciation dictionary.	“s-oh-r-iy ay l-eh-t y-uw d-aw-n ih-f ay l-eh-t y-uw d-aw-n"
original_response	Raw transcription provided by listeners during tests (# = no response).	“Sorry, a let's keep sound, a let's keep sound”
response	Normalized version of original_response (used for scoring).	“sorry a let us keep sound a let us keep sound”
response_pronunciation	Phonemic transcription of the response using the BEEP pronunciation dictionary	"s-oh-r-iy ey l-eh-t ah-z k-iy-p s-aw-n-d ey l-eh-t ah-z k-iy-p s-aw-n-d"
n_words	Number of words in prompt after expanding contractions (see data construction)	10
words_correct	Number of correctly identified words in listening tests	3
correctness	Intelligibility scores: ratio of words_correct to n_words.	0.3
n_phonemes	Number of phonemes in the prompt_pronunciation.	24
phonemes_correct	Number of correctly identified phonemes in listening test	14
phoneme_correctness	Intelligibility score at phoneme-level: ratio of phonemes_correct to n_phonemes	0.583333333
hearing_loss	Indicating if the signal audio has no processing for hearing loss (**No Loss**); **Mild** simulated hearing loss; or **Moderate** simulated hearing loss	“Moderate”

## 4 Experimental Design, Materials and Methods

The CLIP dataset consists of 11,072 music signals taken from popular Western music sung in English, derived from the FMA dataset [1]. Using music likely to be unfamiliar to participants in our listening test, ensured that the intelligibility scores measured lyric perception rather than song familiarity or memory. FMA contains more than hundred thousand tracks across a wide range of musical genres and licenses, and this enabled heterogeneity in the CLIP dataset. Re-purposing FMA for this task required addressing several challenges. First, FMA does not provide ground truth transcriptions of the lyrics. Second, FMA provides a top genre label for each track, however, this is a subjective and unreliable because it is based on sub-genres assigned by whoever uploaded the track and sometimes it is missing due to conflicting sub-genres according the FMA’s hierarchical taxonomy of genres (e.g. ‘experimental pop’ and ‘singer-songwriter’). Third, licences for many tracks do not permit derivative works. Finally, it does not give intelligibility scores.

**Fig. 2:**
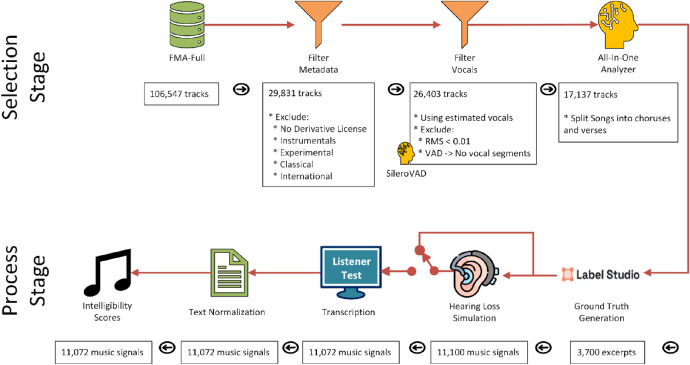
Pipeline of the construction of the CLIP dataset separated into Selection and Process stages.

As shown in Fig. 2, the construction of the dataset can be described in two stages. First, we selected suitable FMA tracks for inclusion in the dataset. Second, we processed these tracks to generate ground truth and human transcriptions in listening tests. A detailed description of each stage is provided below.

### 4.1 Selection stage

The processes were designed to be automatic, to reduce biases that might arise if humans made the selection.

#### 4.1.1 Audio tracks selection

We started with the FMA-Full version of the dataset [1]. This gave 106,574 complete pieces of music. Using the dataset’s metadata, we ran a cascade process to exclude tracks that were not suitable for a lyric intelligibility dataset for Western popular music sung in English.

First, we excluded 42,735 tracks with licenses that did not explicitly allow derivative works because we later applied a hearing loss simulation to the music. Second, using the top-genre label, we filtered out 9184 tracks labelled as either Instrumental, Experimental, Classical or International. Instrumental music should not have lyrics; Experimental and Classical included many tracks that were not Western popular music; and International had many examples not in English. Next, from the tracks without top-genre, we applied the same filtering process to the sub-genre label. This removed a further 22,027 tracks. At this point we had 29,831 tracks.

#### 4.1.2 Filter songs without vocals

The tracks were then analyzed to exclude any for which no vocal activity could be reliably identified. Vocals were first estimated using the HTDemucs music source separation model [6]. For each estimated vocal line, we computed the root-mean-square (RMS) amplitude of the samples in the time domain and also detected vocal segments using the Silero VAD voice activity detection model [7]. We excluded 3428 tracks in which Silero VAD did not detect any voice segments or where the RMS of the estimated vocals was below 0.01 (-40 dBFS). This process reduced the number of tracks to 26,403 tracks.

The application of HTDemucs and Silero VAD to identify music without vocals was not perfect. While HTDemucs is a state-of-the-art music source separation model, it was primarily trained on audio where the mixture is assumed to be a linear combination of all components. This assumption will not hold for all FMA tracks, as some will include non-linear effects applied to the mixture such as mastering compression. This could lead to a mismatch between the separation model and the FMA data. Furthermore, the pre-trained Silero VAD model was not developed for music, rather it was trained and tested on speech in noise. Despite this, the Silero VAD correctly identified vocal segments in most cases, particularly when pauses occurred between different lyric lines or sections. Although using HTDemucs and Silero VAD led to some tracks being incorrectly classified as containing vocals, the errors were detected during the manual ground-truth transcription and the tracks then discarded.

### 4.2 Selecting a chorus or verse

To maximize the diversity of the dataset, we prioritised including short excerpts from as many different songs as possible, rather than using longer excerpts from fewer songs. For this reason, we restricted each track to a single section, choosing either a chorus or a verse. These sections were targeted because they are the most likely to contain vocals. To do this, we used the All-In-One Music Structure Analyzer [8] to detect the different sections within the tracks. Each section is labeled as Intro, Chorus, Verse and Bridge, among other labels. For the CLIP dataset, we randomly select a chorus or a verse section. The All-In-One model detected a chorus or verse in 17,137 tracks.

### 4.3 Process stage

#### 4.3.1 Construction of ground truth lyric transcription

From the 17,137 tracks, only 3500 randomly chosen choruses and verses were transcribed, because this was sufficient for Cadenza ICASSP 2026 challenge. We recruited seven native English-speaking PhD students from the Universities of Salford and Sheffield to do the transcription. Using Label Studio [2], each annotator was assigned 500 random sections (i.e. a chorus or verse) with no overlap between assignments. Three versions of the audio were provided to assist the annotators: (1) the original; (2) the vocals extracted using the HTDemucs musical source separation model, and (3) the music with low-frequencies below 300 Hz removed using a second-order Butterworth high-pass filter. Annotators were instructed to use good quality headphones to facilitate transcription.

Annotators were asked to identify and transcribe all lyric phrases in each section. Lyric phrases were defined as groups of 5 to 10 words forming a meaningful syntactic unit, though not necessarily a complete sentence. They could listen to the tracks as many times as needed and use all three versions to clarify any ambiguities. Additionally, annotators were instructed to: (i) flag any obscene lyrics for removal; (ii) mark phrases where one or two words were unintelligible, and (iii) transcribe numbers and initialisms as they were heard. After transcription, to resolve ambiguous cases published lyrics from websites such as bandcamp.com and genius.com were used if available.

The resulting phrases were then post-processed to ensure data consistency and quality. Using the text transcription, we removed repeated phrases from the same track; excluded phrases where a single word was repeated more than twice; and retained only those containing between five and ten words. Phrases with fewer than five words were concatenated with the following phrase, while phrases with more than ten words were manually reviewed to extract a phrase of 5 to 10 words. The audio was auditioned by members of the Cadenza team, and the boundaries adjusted to include only the transcribed words. This resulted in a final set of 3700 audio excerpts with ground truth transcriptions. These came from 1452 tracks.

A small number of transcriptions (34 excerpts) include words marked as “?”, indicating that they were unintelligible even to the transcribers under optimal listening conditions. We retained these cases because they reflect the unintelligible phrases that are often present in sung language.

#### 4.3.2 Hearing loss simulation

Intelligibility scores were generated using listening tests. We first split the excerpts by artist into training, validation and evaluation using a split of 80 %, 10 % and 10 %, respectably. Each excerpt was processed using a hearing loss simulator [9] for mild and moderate hearing loss severity [10]. The simulator uses a gammatone filterbank model of the auditory periphery to models four main aspects of hearing loss: decreased audibility and raised auditory thresholds; reduced dynamic range and loudness recruitment; loss of temporal resolution, and loss of frequency resolution due to loss of cochlear hair cell sensitivity. To apply the hearing loss simulator, an audiogram corresponding to the appropriate severity was randomly selected. Different sets of audiograms were used for each split, drawn from the audiogram pool of the Cadenza CAD1 challenge [11]. Symmetric hearing loss was applied by using the same audiogram left and right. We did not include severe or profound hearing loss as those listeners are more likely to be using hearing devices. The hearing loss simulation was implemented in Python using the PyClarity module [12]. Before applying the simulation, excerpts were normalized to 75 dB SPL. After processing, gains were applied to the music signals so they all had the same RMS value. This was done to prevent large loudness differences between samples in the listening tests arising from the different hearing loss severities. It also better reflects real scenarios, where listeners would set volume levels allowing for their hearing acuity. Finally, the signals were peak normalized using the largest absolute peak value in all music signals.

This yielded 11,100 music signals in total, with each excerpt having three versions: the original signal extracted from the FMA dataset, one with simulated mild hearing loss, and one with simulated moderate hearing loss.

#### 4.3.3 Listening tests to generate lyric intelligibility scores

We shuffled all 11,100 music signals and divided them into 111 groups of 100 music signals each, with each group assigned to a different listener for annotation. Each group could contain music from the training, validation, and evaluation splits. This grouping was designed to help normalize listener behaviour across all splits. Additionally, we ensured that no two versions of the same excerpt appeared in the same group e.g. a listener did not hear the same excerpt with no loss and moderate hearing loss. This was done to avoid potential overestimation of intelligibility caused by a listener hearing the same lyrics more than once. All groups had a similar number of no loss, mild and moderate music signals.

Listening tests were carried out online using Prolific (www.prolific.com) in the second half of July 2025. We recruited adults aged 18 to 40 who were native speakers of English and self-reported no hearing difficulties. The experiment had a median duration of 42 min; each experiment had a cost of £9.80 distributed as £7.00 participant reward plus platform fee and VAT. In the experiments, each music signal was looped to play exactly twice, with the first playback intended to help listeners adapt to the genre and vocal style. Listeners were instructed to type what they heard, and if they were unable to identify any lyrics, they were asked to enter “xxx”.

To assess the quality of the responses, we analyzed the number of “xxx” submissions per participant. Since an “xxx” response indicates zero intelligibility, it was important to confirm that these cases reflected genuinely unintelligible signals rather than careless low-effort responses or limitations in reproduction equipment. Across all participants, there were 2007 “xxx” responses, with a mean of 18 per participant (SD = 14). Inspection of the distribution revealed a small group of 13 participants who each submitted 40 or more “xxx” phrases, far above the typical range. These 13 participants accounted for 624 “xxx” responses, with an average of 48 per participant. We excluded these data and re-ran the experiments with new participants. The replacement experiments produced 253 “xxx” responses, averaging 19 per participant. After this replacement, the total number of “xxx” responses decreased from 2007 to 1636, and the mean per participant dropped from 18 to 17. The participant demographics were: 50.5 % female and 45.5 % male; mean age=31.0, standard deviation (SD)=5.7, *N* = 111; and simplified ethnicity 6.3 % Asian, 22.5 % Black; 6.3 % Mixed and 64.9 % White.

After the listener test, we conducted a quality-control pass in which each excerpt was reviewed individually by listening to the audio, examining its corresponding transcription, and analysing listener test participants’ feedback. During this stage, 28 responses were flagged and subsequently discarded due to errors encountered during data collection. These issues included: (i) segments with incorrect boundaries; (ii) audio files that failed to load during the listening test, as reported by participants; (iii) empty participant responses, possibly due to connection errors, and (iv) repeated songs resulting from multiple submissions of the same track on the FMA dataset. This resulted in a final dataset of 11,072 music signals with a duration ranging between 1.1 and 22.9 s with an average of 4.5 s and standard deviation of 2.0 s.

#### 4.3.4 Intelligibility scores

Intelligibility scores were computed as the ratio of matching words between the ground truth transcription (prompt) and the participants’ responses in the Prolific listening tests (response). Before computing these scores, text normalization was applied to both prompts and responses, following processes used in speech intelligibility research:

• Convert all numbers to words (e.g. 1.6 = one point six).
• Remove punctuation, retaining only contraction marks.
• Correct obvious misspellings (e.g. WASNT = WASN’T, AWNSER = ANSWER).
• Expand contractions (e.g. I’M = *I* AM).
• Convert to lower case.

For the prompts, text was normalized and expanded to its maximum form. For example, the 9-word sentence “I don’t know if I’ll go to the party!”, was expanded to the 11-word form “i do not know if i will go to the party”. In cases of contraction ambiguity, such as where’d (which could expand to where did or where had), one option was selected at random. For responses, on the other hand, all possible combinations were considered.

To avoid penalizing the use of different homophones between prompts and responses, both were transcribed into their phonemic forms using the BEEP pronunciation dictionary [13]. To maintain word-level computations, all phonemes within a word were concatenated using a dash. For example, the word “don’t” was represented as “d-uw n-oh-t". This method allowed us to compare words based on their phonemic content, avoiding spelling or homophone discrepancies, while still computing intelligibility at the word level. For words that were not obvious misspellings, phonemic forms were generated to complement the BEEP dictionary by using a grapheme-to-phoneme model trained with Phonetisaurus [14] and the BEEP dictionary.

In cases where the BEEP dictionary provides multiple phonemic transcriptions for the same word, one random alternative was selected for the prompt transcription, and all alternatives were evaluated for the responses, keeping the one that resulted in the highest score. For example, for the word LEAD, the BEEP dictionary provides two transcriptions: l-eh-d (the metal) and l-iy-d (to guide).

A dynamic programming algorithm is used to find the alignment that minimizes the Levenshtein distance between the ground truth and the listener transcription. This alignment allows us to count how many ground truth words were correctly recognized. This was implemented using the jiwer Python module [15].

Following the same approach from Sharma and Wang [16], the final intelligibility score was defined as the maximum number of matching words between the prompt and all available response alternatives. For example, if a prompt contains five words and there are three alternative phonetic transcriptions of the response, where the first has two matches, the second has one match, and the third has three matches, the final score is calculated as the ratio three over five.

### 4.4 Analysis of the dataset

**Fig. 3:**
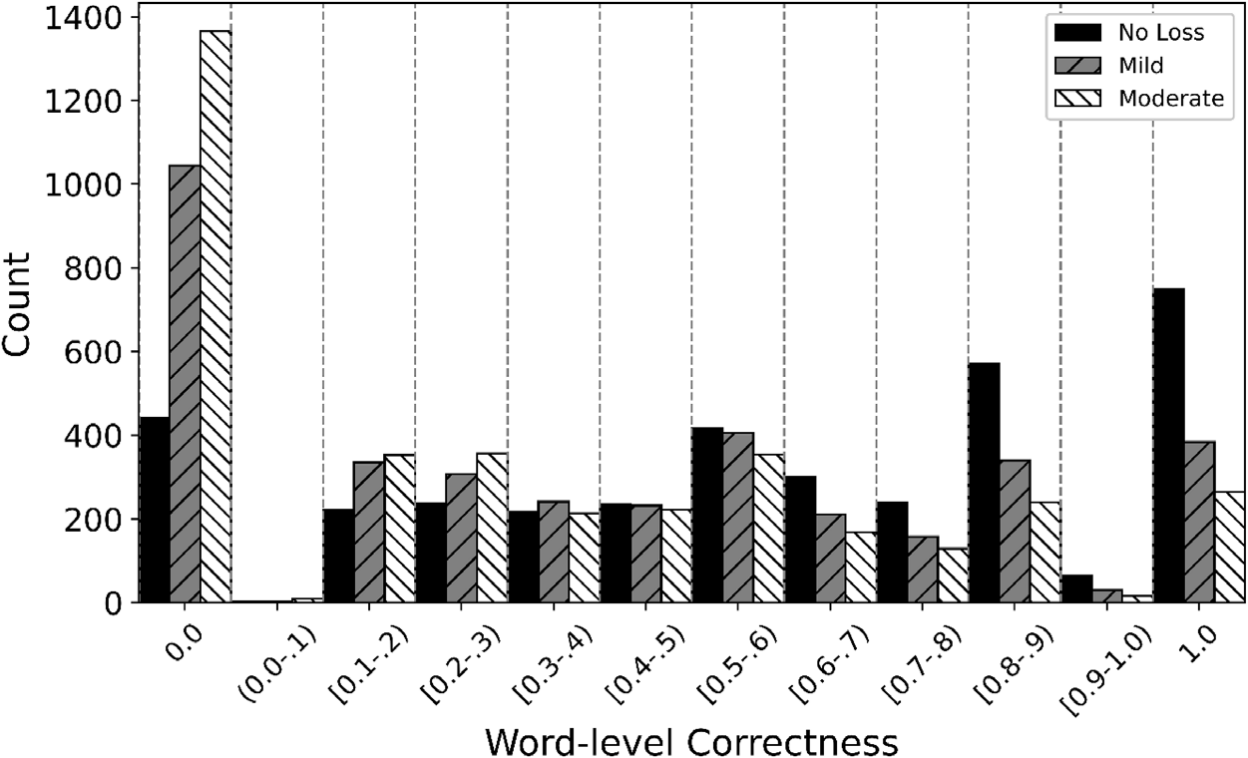
Distribution of correctness score (intelligibility at word-level) per hearing loss severity for the whole dataset.

Overall, the training, validation, and evaluation sets have similar distribution of intelligibility scores. The Kolmogorov-Smirnov test showed no significant difference between training-validation (D(8802, 1175)=0.0298, *p* = 0.34), training-evaluation (D(8802, 1095)=0.0295, *p* = 0.48), and validation-evaluation (D(1,1175, 1095)=0.0247, *p* = 0.87) sets. Fig. 3 shows the distribution of the intelligibility scores (correctness) across hearing-loss severity groups. The Kolmogorov-Smirnov test shows significantly different distributions for No Loss-Mild (D(3690, 3691)=0.2221, *p* < 0.001), No-loss-Moderate (D(3690, 3691)=0.3212, *p* < 0.001) and Mild-Moderate (D(3691, 3691)=0.1089, *p* < 0.001) comparisons. Note that the correctness score was computed as the ratio of correctly identified words in phrases containing between 5 and 10 words. Consequently, only a discrete set of score values is possible.

The data are skewed toward scores of 0.0 and 1.0. In the training dataset, there are, in total, 2849 samples with an intelligibility score of 0.0, with fewer samples in the No Loss group compared to the Mild and Moderate groups. In contrast, samples with an intelligibility score of 1.0 (1398 in total) show the opposite trend, with more samples in the No Loss group than in the Mild and Moderate groups. This pattern is expected, as the Moderate group represents the most challenging listening scenario.

**Fig. 4:**
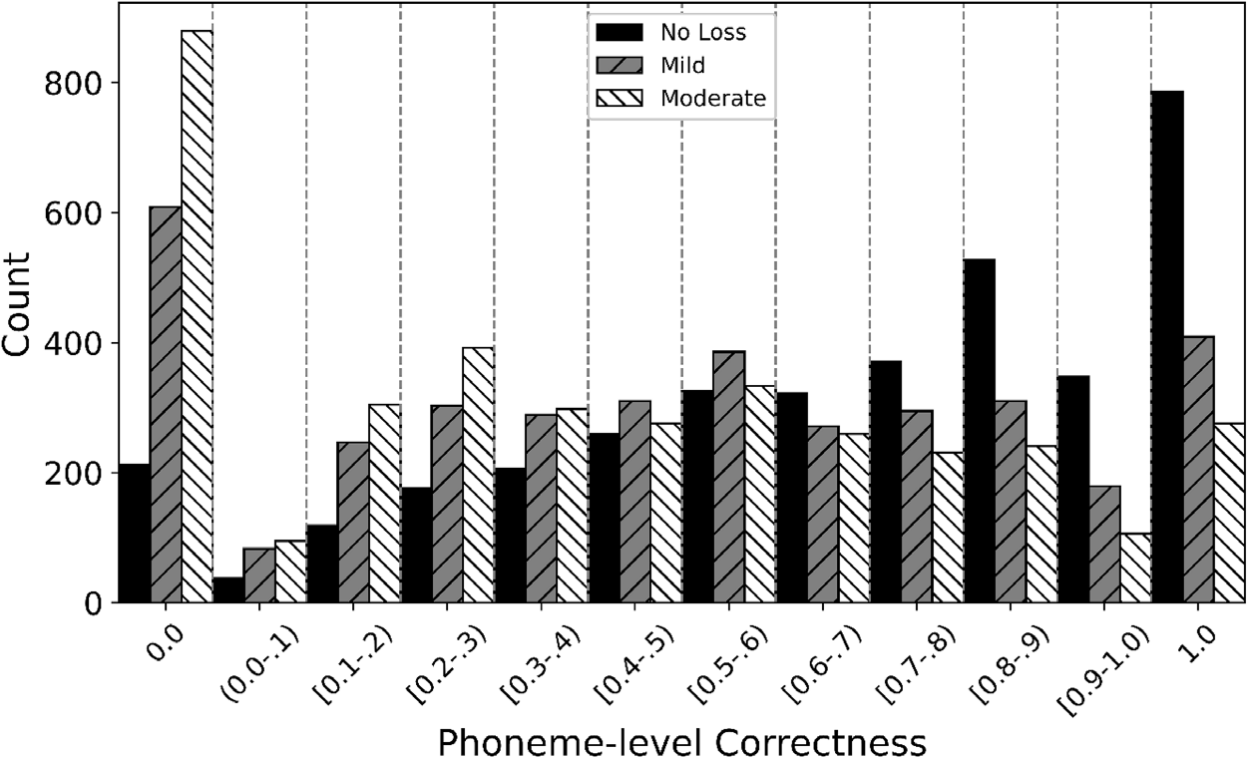
Distribution of phoneme-level correctness per hearing loss severity for the whole dataset.

To further analyze the dataset, we used the phonemic transcriptions to compute the ratio of correctly identified phonemes (phoneme-level correctness). The distributions between training, validation and evaluation sets are similar. The Kolmogorov-Smirnov test showed no significant difference between distributions (training-validation (D(8802, 1175)=0.0350, *p* = 0.18), training-evaluation (D(8802, 1095)=0.0321, *p* = 0.23), and validation-evaluation (D(1,1175, 1095)=0.0327, *p* = 0.56). Fig. 4 shows how the distributions vary with hearing loss severity, with Kolmogorov-Smirnov tests indicating significant differences for the No Loss-Mild (D(3690, 3691)=0.2478, *p* < 0.001), No-loss-Moderate (D(3690, 3691)=0.3445, *p* < 0.001), and Mild-Moderate (D(3691, 3691)=0.1230, *p* < 0.001).

The number of samples with zero intelligibility decreases to 1700, while the number of samples with an intelligibility score of 1.0 increases to 1471. This increase in the number of cases with an intelligibility score of 1.0 is expected because the correctness metric does not penalise transcription errors, and a phoneme-level metric rewards partially correctly words. For example, the prompt “when I walk by I see” and the response “when I walk by ice cream” have the phonemic transcriptions “w-eh-n ay w-ao-k b-ay s-iy” and “w-eh-n ay w-ao-k b-ay s k r iy m”, respectively. At the word level, the response is correct for 4 out of the 6 words in the prompt (misidentifying the words “I see”), resulting in a word-level correctness of 0.67. However, at the phoneme level, the phonemes for “I see” (ay s-iy) are all present in the phonemic transcription of “ice cream”, resulting in a phoneme-level correctness of 1.0.

**Fig. 5:**
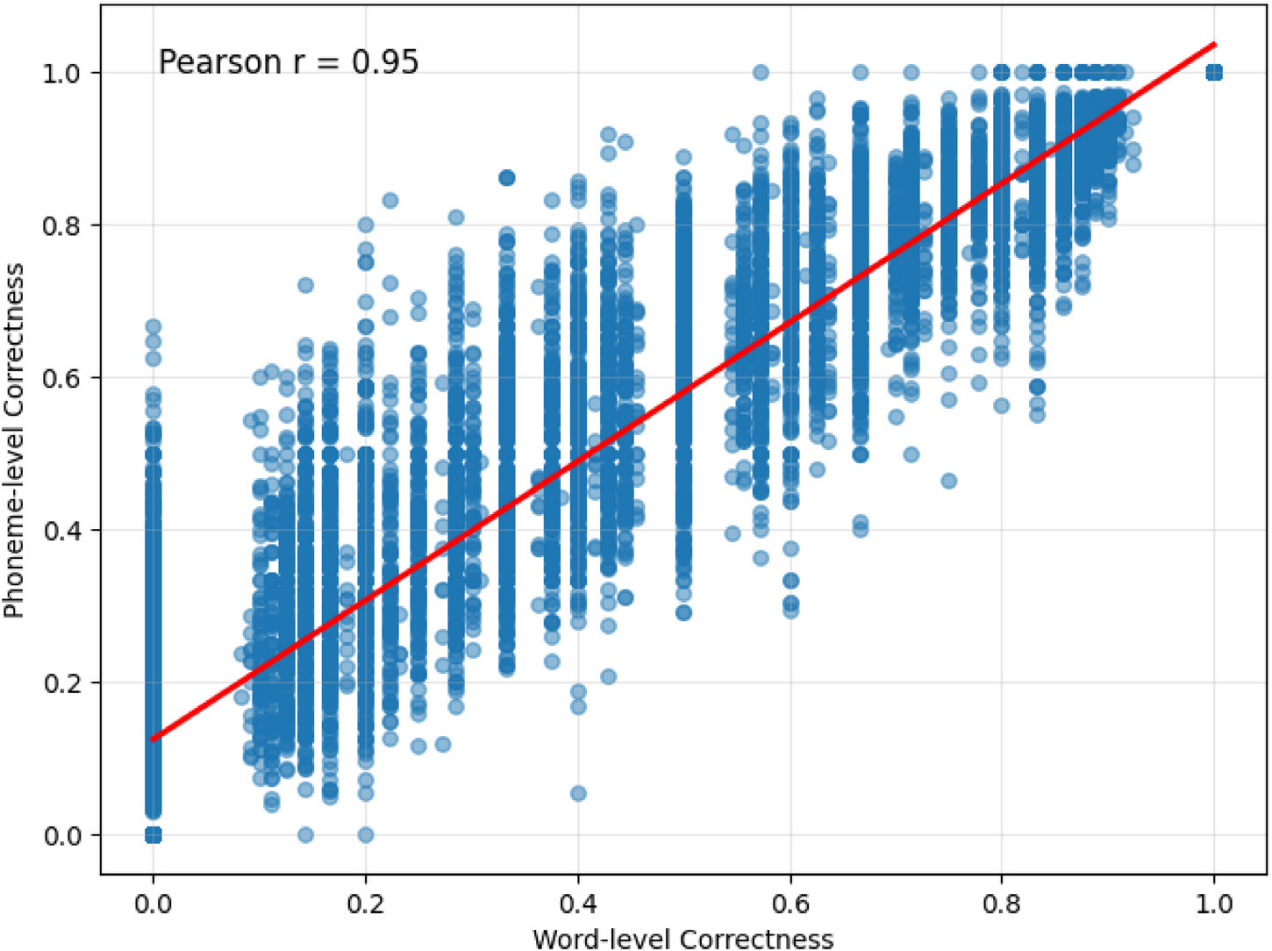
Scatter plot between phoneme-level intelligibility and word-level correctness. There are 1078 datapoints at (1,1).

To quantify the impact of using word-level versus phoneme-level correctness, we examined the differences between the scores. Fig. 5 shows a scatter plot between word-level correctness and phoneme-level intelligibility showing a Pearson correlation coefficient of 0.95. The average difference is 0.09 (standard deviation 0.11, *N* = 11,072). The difference between both scores is statistically significant (*t*-test, *t*=-78.6, *p* < 0.001, df=11,071; medium effect size d: 0.75).

## Limitations

Since most songs in the FMA dataset do not have their lyrics published online, ground truth transcriptions had to be manually generated by human annotators. Despite the annotators’ efforts to produce high-quality transcriptions, some errors may remain due to mishearing, and consequently the ground truth can therefore be considered somewhat noisy.

Intelligibility datasets typically collect multiple responses per audio signal and compute an average score. In the CLIP dataset, however, each segment has only a single intelligibility score from one participant. Collecting multiple scores per segment is costly and, where resources are limited, results in small datasets; for example, Sharma and Wang [16] only used 200 song excerpts. The primary objective of this dataset was to evaluate lyrics intelligibility prediction algorithms that use machine learning across the entire set, rather than predicting the exact intelligibility of each music sample. Consequently, we prioritised covering a wide range of music rather than collecting repeated responses to the same material. As we are using the data to compare prediction algorithms across music samples, this implicitly includes averaging over annotators, but not on a sample-by-sample basis. The consequence of this is that our dataset has a noise-floor due to the variance between annotators.

## Ethics Statement

The authors have read and followed the ethical requirements for publication in Data in Brief. All procedures were approved prior to testing by the North east - Newcastle & North Tyneside 2 Research Ethics Committee (23/NE/0071)

## Credit Author Statement

**Gerardo Roa-Dabike**: Conceptualization, Methodology, Software, Data Curation, Visualisation, Writing -Original Draft, Writing - Review & Editing; **Trevor J. Cox**: Conceptualization, Methodology, Writing - Review & Editing, Supervision, Funding acquisition; **Jon P. Barker**: Conceptualization, Methodology, Writing - Review & Editing, Supervision, Funding acquisition; **Bruno Fazenda**: Conceptualization, Funding acquisition; **Simone Graetzer**: Conceptualization, Funding acquisition, Writing - Review & Editing; **Rebecca R. Vos**: Conceptualization; **Michael A. Akeroyd**: Conceptualization, Funding acquisition; **Jennifer Firth**: Conceptualization; **William M. Whitmer**: Conceptualization, Methodology, Funding acquisition, **Scott Bannister:** Conceptualization; **Alinka Greasley**: Conceptualization, Funding acquisition;

## Data Availability

ZenodoThe Cadenza Lyric Intelligibility Prediction (CLIP) Dataset (Original data).
